# Real-Time Dynamics of Water Transport in the Roots of Intact Maize Plants in Response to Water Stress: The Role of Aquaporins and the Contribution of Different Water Transport Pathways

**DOI:** 10.3390/cells13020154

**Published:** 2024-01-15

**Authors:** Maksim Suslov, Amina Daminova, Juluskhan Egorov

**Affiliations:** Kazan Institute of Biochemistry and Biophysics, FRC Kazan Scientific Center, Russian Academy of Sciences, P.O. Box 30, Kazan 420111, Russia

**Keywords:** water transport in roots, transpiration, apoplast, symplast, cell water permeability, aquaporins

## Abstract

Using an original methodological and technical approach, we studied the real-time dynamics of radial water transfer in roots and transpiration rate in intact maize plants in response to water stress. It was shown that the response of maize plants to water stress, induced by 10% PEG 6000, was accompanied by changes in the intensity and redistribution of water transfer along different pathways of radial water transport in the roots. It was shown that during the first minutes of water stress impact, the intensity of transcellular and symplastic water transport in the roots decreased with a parallel short-term increase in the transpiration rate in leaves and, presumably, in apoplastic transport in roots. Further, after a decrease in transpiration rate, the intensity of transcellular and symplastic water transport was restored to approximately the initial values and was accompanied by parallel upregulation of some PIP aquaporin genes in roots and leaves, changes in aquaporin localization in root tissues, and changes in xylem sap pH. Under water stress conditions, cell-to-cell water transport in roots becomes dominant, and aquaporins contribute to the simultaneous regulation of water transport in roots and shoots under water stress.

## 1. Introduction

According to modern concepts, the plant hydraulic system (water transport system) provides regulation of water transfer to support the optimal water balance in different parts of the plant necessary for normal growth and development. The availability of water and the efficiency of its use by plants depend, on the one hand, on the water content of the environment and, on the other hand, on the development and functioning of the plant hydraulic system, and also on the ability of this system to respond rapidly to environmental changes [1,2]. The influence of drought and other abiotic factors that cause water stress results in noticeable changes in the plant hydraulic system, expressed, for example, in changes in root, stem, and leaf conductance; changes in stomatal conductance; and in the architecture of separate components of the hydraulic system [3,4,5]. In addition, the character of these changes may depend on the dynamics and duration of the stress [6]. The plant root system is the primary target during the water stress caused by a water deficit. Root hydraulic conductance is known to change to varying degrees under water deficit conditions [7]. However hydraulic conductance is an overall property that depends on the contribution of different water transfer pathways in the roots. According to the composite transport model, there are three parallel pathways of water and solute transport in roots: apoplastic (water passes along the cell walls), symplastic (water moves from cell to cell through plasmodesmata), and transcellular (water moves between cells and cell membranes) [2,8]. It should be noted that it is technically difficult to separate the contribution of symplastic and transcellular pathways to the total water transfer. Therefore, symplastic and transcellular pathways are often combined into one cell-to-cell pathway. Based on the evaporative demand of the shoot, the composite transport model would allow for an adjustment between the apoplastic, symplastic, and transcellular pathways [2]. Water transport pathways may differ in hydraulic conductivity, and the extent to which each pathway contributes to root water channeling in different species, genotypes and in response to the environment, as much as the three pathways can be distinguished, is still not fully understood [9,10]. A water deficit is known to result in the redistribution of water transfer pathways’ contribution in roots. The contribution of the apoplastic water transfer pathway is supposed to dominate under the conditions of sufficient water content and high transpiration rate. Under the conditions of a water deficit, the transcellular pathway, which can be rapidly switched up or down as needed, becomes dominant [11]. The main regulatory function then belongs to aquaporins (AQPs). Studies on plant AQPs and plant water relations have been carried out for many years and have led to the discovery of diverse functions performed by AQPs [12]. These proteins have been shown to be involved in root conductance and, therefore, water uptake and transport by roots [13]. AQPs have also been implicated in the regulation of stomatal opening/closing and carbon dioxide penetration in leaves, thereby affecting the processes of transpiration and photosynthesis [14,15,16,17]. Furthermore, the involvement of AQPs in stem conductivity has been documented [18]. Many studies have demonstrated the significant role of AQPs in the acquisition of abiotic stresses tolerance [19]. For example, water stress can induce changes in the level of gene expression and number of AQPs in plant roots and affect the hydraulic conductivity [20]. Changes in AQP gene expression in response to water stress can also occur in leaves [21,22]. Obviously, the effect of water stress cannot be limited to responses in root hydraulics. Changes in root hydraulic parameters are bound to cause changes in leaf hydraulic parameters and vice versa. Recently, an increasing number of studies have focused on the interaction and coordination between different components of the plant hydraulic system, especially during abiotic stress [7,23,24,25]. A functional relationship between stomatal conductance and leaf, stem and root hydraulics has been demonstrated [26,27,28,29]. However, to date, the dynamics and interactions between water transport processes in the roots and shoots of intact plants under water stress and the role of AQPs in this have not been fully studied.

Therefore, the aim of the present work is to study the real-time dynamics of water transport in roots, including the estimation of the response of transcellular, symplastic, and apoplastic pathways; leaf transpiration rate; and other physiological parameters in intact maize plants directly under the influence of water stress. The original technical approach was used based on the NMR spin-echo method with paramagnetic doping and fluorescent nanoparticles [30,31]. This work is focused mainly on early response to water stress and the role of AQPs in roots and leaves.

## 2. Materials and Methods

### 2.1. Plant Material

**Figure 1:**
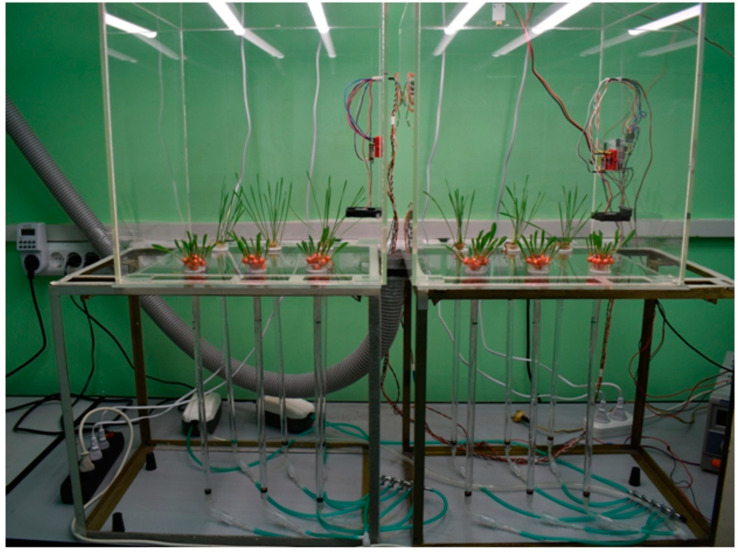
Climatic plant growth chambers with control of temperature, humidity, and carbon dioxide concentration with the possibility of interfacing with NMR equipment to study the state of the plants’ hydraulic systems under controlled stress and environmental conditions.

Maize plants (var. Mashuk) were grown hydroponically in aerated plastic tubes for 7 days after germination before starting the experiments. Plants were grown in climate chambers under a 12-h photoperiod (irradiance of 15 W m^−2^) with temperature, humidity, and atmosphere carbon dioxide concentration control (Figure 1). Before transplanting plants after germination into tubes, the lateral roots of the plants were removed. During plant growth, the temperature in the chambers was maintained within 24–25 °C during the day and 20–21 °C at night. Air humidity in the chambers was 45–50%, and the carbon dioxide concentration was 380–420 ppm. In total, about 150 plants were grown simultaneously in the climate chamber. They were distributed in different test tubes. This allowed for measurements of transpiration rate and root and shoot growth rate to be made simultaneously with the NMR measurements. In parallel, parts of the plants was fixed to analyze the levels of AQP gene expression and AQP localization, and samples were taken to measure the pH of the xylem sap and the relative water content in the leaves.

### 2.2. NMR Measurements and Technique

To provide the necessary signal-to-noise ratio and achieve biological variability averaging, each sample tube for NMR experiments contained 7-day-old intact maize plants with approximately equal root length. Before each NMR measurement of diffusional and relaxation parameters of water transport in plants, one of the climate chambers with one sample tube was installed on the NMR relaxation-diffusion meter so that part of the root absorption zone was inside the NMR probe. All NMR measurements were carried out at the absorption zone about 3–4 cm from the root tip. During a certain step, after measurement of the control values of water transport in the roots, the roots were treated with water stress caused by a change of the water solution in which plants grew (CaCl_2_ 0.0025 M) by the addition of a 10% solution of polyethylene glycol (PEG 6000). Then, the parameters of water transport in intact plant roots immediately under the effect of water stress were dynamically measured for a long period of time. Thus, this approach allowed for the registration of both early and late responses to water stress. NMR measurements were performed in triplicate.

The experiments were performed using a gradient spin-echo NMR relaxation-diffusion meter with magnetic field frequency of 20 MHz and with digital radiofrequency pulse generation complex “Spin Track” (Resonance Systems Ltd., Yoshkar-Ola, Russia). In the diffusion measurements, a stimulated echo sequence (90°-90°-90° radiofrequency pulses) with a pulsed magnetic field gradient of up to 2 Tl/m was used [32,33]. The magnetic field gradient was applied perpendicular to the root segments, i.e., diffusion measurements were made in the radial direction of the roots. Commonly, the 3-fold accumulation of magnetization signals was used with a 2-step phase cycling of r/f pulses and, correspondingly, reference voltage on the phase detector. The recording of diffusion decays (DDs) of water magnetization involved measuring the relative amplitude of stimulated echo signals (R = A(g)/A(0)) against the amplitude of magnetic field gradient pulses, g, and pulse duration, δ, while varying the inter-pulse interval, td (diffusion time). The quantification of diffusion transport was based on the average diffusion coefficient, which is independent of the diffusion coefficient distribution. The formalism of the effective diffusion coefficient (D_ef_) was used [34]. The value of the average D_ef_ was determined from the relation:

(1) $$R=exp({-\gamma }^{2}\delta^{2}g^{2}t_{d}D_{ef}),$$

by the slope of the initial part of the DD, according to the condition:

(2) $$D_{ef}={\left.\frac{\partial \left[\ln R(g)\right]}{\partial \left[-\gamma^{2}\delta^{2}g^{2}\left(t_{d}-\delta /3\right)\right]}\right|}_{g\to 0},$$

It is worth noting that water transport in plants (including stressed plants) has been analyzed non-invasively using NMR in many studies [35,36]. However, in these studies, the NMR method was more adapted to NMR imaging and provided information on water distribution in plant tissues and organs, the water content of tissues in stems, and water distribution in the axial direction of primary and lateral roots. This method also allows for estimating the rate of axial water fluxes, but mainly in large stems. The disadvantage of this method is the low resolution limited to the tissue level. In contrast to the above-mentioned works, in our study, the spin-echo NMR method with a pulsed magnetic field gradient was adapted to measure diffusive water transport specifically in the radial direction of the roots and allowed us to obtain information on the water permeability of cells. The use of paramagnetic doping to contrast the symplastic pathway of water transport in the root is also original.

### 2.3. Determination of the Effective Permeability Coefficient P

To determine the effective coefficient of intercellular water permeability (P) from the value of the average Def, the Crick relation [37] was used, which is obtained by considering the resistance to the passage of water molecules through a series of parallel permeable barriers separated by a distance a:

(3) $$\frac{1}{D_{\infty }}=\frac{1}{D_{0}}+\frac{1}{Pa},$$

There is no reference to the details of intercellular transport mechanisms in relation (3). The values of the diffusion coefficients D_0_ and D_∞_ are determined from the experimental dependence of the measured diffusion coefficient on the diffusion time t_d_ [34]. This dependence consists of three regions: the region of free diffusion, where the self-diffusion coefficient is constant and close to the bulk water self-diffusion coefficient; the region of restricted diffusion, where there is no averaging of local translational motions of water molecules within a sufficiently large volume; the region of hindered diffusion, which depends on the intercellular permeability, and this dependence leads to diffusion averaging at distances exceeding the pore (cell) size [34]. In this region, the water self-diffusion coefficient D_∞_ is also independent of the diffusion time t_d_. It was shown in preliminary experiments that for maize roots, region of hindered diffusion corresponds to diffusion times of 600–700 ms and more.

The characteristic size of restrictions, which is necessary for calculations and which is commonly associated with the cell size, can be determined using the Einstein–Smoluchowski relation:

(4) $$D_{s}\left(t_{d}\right)=\frac{a^{2}}{{6t}_{d}},$$

with diffusion coefficients, measured under the conditions of totally restricted diffusion, when the measured diffusion coefficient is a linear function of the inverse diffusion time (t_d_^−1^). However, when the restricting barriers are permeable, this dependence is distorted, and consequently, the impact of the permeability on the dependence D_s_(t_d_) should be excluded. This task has been solved within the frames of the so-called scaling approach, consisting of renormalization (scaling) of the experimental dependence $D_{s}\left(t_{d}\right)$ into D_ef2_(t_d_) [31,38]. Scaling for long t_d_ where $D_{s}\left(t_{d}\right)\to D_{\infty }\ll D_{0}$ is given by:

(5) $$D_{ef2}\left(t_{d}\right)=D_{0}\frac{D_{s}\left(t_{d}\right)-D_{\infty }}{D_{0}-D_{\infty }},$$

Because t_d_ is limited by the relaxation time, the value of D_∞_ often cannot be determine directly from the experiment. When renormalized, D_∞_ is positioned as a fitting parameter, deriving the experimental dependence into the dependence D_ef_(t_d_^−1^). 

### 2.4. Estimation of Water Transport Selectively along the Plant Root Symplast

One of the weak points of the composite model of water transport in roots and its modified version to date, MECHA (Model of Explicit Cross-Section Hydraulic Anatomy) [39], is the lack of experimental data on the plasmodesmata hydraulic conductivity. It results from the lack of the necessary methodical and technical approaches to the measurement of water flow along the symplast system through plasmodesmata. In the present work, we carried out measurements of the water translational diffusion selectively along the root symplast using the NMR spin-echo method with pulsed magnetic field gradient. Selectivity of water transfer along the symplast system was achieved using the effect of relaxational suppression of the extracellular water NMR signal by penetration of paramagnetic particles with high relaxation efficiency (paramagnetic doping) into the root intercellular space. The essence of the approach is that a paramagnetic complex penetrates into the root intercellular space but not into the cells, accelerating the process of magnetic relaxation of the extracellular water and thus excluding the contribution of the extracellular water into the registered NMR spin-echo signal decay. As a result, NMR control of the translational diffusion is applied to the signal from water moving along the symplast through plasmodesmata unavailable for paramagnetic complexes Mn^2+^-trans-1,2-diaminocyclohexane-N,N,N′,N′ of tetraacetic acid (MnDCTA). Earlier, we showed that this is the most efficient contrasting agent. Anisimov and Suslov [31] described in detail the application of paramagnetic doping and the NMR spin-echo method to contrast the symplastic water transfer in plant roots. Thus, in the study of the water stress effect on the symplastic water transfer in roots, the intact plant roots were incubated for one hour in the MnDCTA (pH 6.5) 10 mM solution before the roots’ treatment with a PEG 10% solution. The degree of the paramagnetic complex penetration into the root apoplast was controlled by the dynamics of the extracellular water spin–spin relaxation time and by the mono-component character of the magnetization diffusional decay of the sample water measured in the course of incubation of the intact roots in the paramagnetic solution. After 60–70 min of incubation, the paramagnetic complex filled the extracellular space. Then, the control measurement of root water self-diffusion coefficients was carried out. After that, the paramagnetic solution was replaced by a 10% PEG solution in 10 mM MnDCTA. Thus, under the effect of PEG, the paramagnetic solution remained in the extracellular space and in the external medium. Immediately before the next measurement of the diffusional decay, which lasted approximately 4 min, the solution of PEG was dropped below the measured zone. After the measurement, the level of the PEG solution in the sample tube was returned to the initial level. In the same way, 20 min later, the next measurement was carried out. This approach allowed for the study of the symplast water transfer dynamics at the early stage of water stress.

### 2.5. Estimation of Relative Contribution of Apoplastic Pathway to Radial Water Transport in Roots under Water Stress

The estimation of the relative contribution of the apoplastic pathway to the radial water transfer in roots under water stress was carried out using two methods. The first one was to compare the rate of decrease in the extracellular (apoplastic) water spin magnetic relaxation in the course of intact plant roots incubated in the MnDCTA solution, which does not penetrate into cells. The point of the method is that the higher the intensity of the apoplast water transfer in plant roots, the faster the paramagnetic agents penetrates into the root apoplast, and, consequently, the higher the rate of the decrease in the spin–spin relaxation time (T_2_). Thus, the rate of T_2_ decrease indicates the intensity of the apoplastic water transport in roots. Intact plant roots were used in the experiment. During the experiment, a sample tube with control or treatment plants was placed into the NMR probe without removing the plants from the growth chamber, and consequently, without disturbance of the climate characteristics. At the same time, the root absorption zone was adjusted in the measuring region of the NMR probe. Then the water solution of MnDCTA 10 mM with a pH of 6.5 was inserted into the root medium through the aperture in the lower part of the sample tube using a syringe. Immediately after that, the relaxational decay of the sample water was registered over one hour in 5-min increments. Sample water magnetization decay was obtained using the Carr–Purcell sequence, and T_2_ was calculated.

The second method was based on the use of the fluorescent mark in the form of fluorescent silicate nanoparticles, which are distributed over the root apoplast and do not penetrate into cells in the course of incubation of the plant roots. The point of this approach is to compare the depth of penetration of nanoparticles into the roots of intact control and treatment plants at the same time that the plants are being incubated in a nanoparticle solution. For control plants, 6 g/L of the water solution with nanoparticles was used. In the experiment with water stress, the same concentration of nanoparticles in the 10% PEG solution was used. Thus, the penetration of nanoparticles took place directly during the water stress influence on plants. In the meantime, the plants were kept in the climate chambers without environmental changes. After 45 min of incubation, the control and experimental plants were removed from the chambers and rinsed in clean water for three minutes. Then, at a distance of 3 cm from the root tip, cross-sections were obtained manually using a razor blade. Fresh cuts were viewed through a confocal microscope LSM780 (CarlZeiss, Jena, Germany).

### 2.6. Transpiration Measurements

The transpiration rate in intact plants was measured by the weighting method on the portable digital weighting scale Ohaus PA214C within the accuracy of 0.0001 g (Ohaus Corporation, Parsippany, NJ, USA). For this purpose, the mass of sample tubes with the three control (without water stress) plants and three plants under the effect of water stress, located in climate chambers or in the NMR probe, was measured every 10 min. Thus, the dynamics of changes in the sample tube mass due to water evaporation from the leaf surface showed the response of transpiration to the addition of PEG into the root medium. Then, the plants were photographed, and the obtained imaged were analyzed using MacBiophotonics ImageJ 1.43m to measure the leaf surface area. The transpiration rate was determined as the mass of evaporated water per leaf surface area unit per time unit.

### 2.7. RNA Extractions, cDNA Synthesis, and PCR Reactions

RNA extractions were performed using primary root absorption zone segments from plants grown in the climate cambers. The samples were frozen in liquid nitrogen at approximately 12 am after being cut. Root segments that were 2 cm long from the absorption zone, 3–4 cm from the root tips, and the middle part of the leaf, which was 2–2.5 cm long, were used. PEG treatment time points were 2 h and three days after the beginning of root treatment with PEG. For each biological replication, root and leaf segments with a total mass of approximately 100 mg were used from three different plants. RNA extractions were performed using the RNeasy Plant Mini Kit following the manufacturer’s instructions (Qiagen, Hilden, Germany). RNA yield and purity were estimated using the Nanodrop TM1000 spectrophotometer (Thermo Fisher Scientific Inc., Waltham, MA, USA). RNA integrity was verified on agarose gels. cDNA synthesis and PCR reactions were performed according to the protocols described in [40]. Each control and PEG treatment time point was analyzed with three technical and three biological replicates. Plasma membrane-localized aquaporins of maize plants likely to transport water were selected according to literature data and additionally verified using the Phytozome13 and UniProt databases. Primers for 11 target and 3 reference genes (Table S1 in Supplementary Materials) were designed using CLC Genomics Workbench 8 Software and synthesized by Evrogen (Moscow, Russia). For normalization in the qPCR analysis, the normalization factor (NF) was used. NF was calculated based on the geometric mean of three selected reference genes.

### 2.8. In Situ Immunolocalization

The study of aquaporin localization in the root tissues of maize plants was carried out based on the protocol used by Hachez et al. [41] with minor changes. Root sections 60 µm thick at a distance of 3–4 cm from the root tip were prepared using the vibratome Leica VT 1000S (Leica Biosystems, Wetzlar, Germany). Sections were incubated for 10 min in a 4% (*w*/*v*) paraformaldehyde solution made in 0.2 M of phosphate-buffered saline (PBS, pH 7.2). Permeabilization of the samples was achieved by dipping the sections for 10 min in 0.25% Triton X-100 in PBS. The sections were incubated for 1 h at 37 °C in PBS containing 5% BSA (blocking solution), then for 1 h at 37 °C with anti-PIP antiserum diluted 1/100 in a blocking solution. Commercial antibodies to PIP2 and PIP1 aquaporins produced by Agrisera (catalogue number AS09 506, AS09 489) were used. After 3 × 20 min washes in the blocking solution, the sections were incubated for 1 h in the dark at 37 °C with fluorescein isothiocyanate (FITC)-conjugated antibodies (dilution of 1/100 in the blocking solution). The slides were then rinsed for 4 × 5 min in PBS and observed using a Leica DM1000 epifluorescence microscope (Leica Biosystems, Germany) fitted with a mercury lamp and appropriate filter cubes (excitation filter 460–500 nm, barrier filter 512–542 nm) for fluorescein isothiocyanate (FITC)-conjugated antibodies. The controls included detection without incubation of sections with the primary antibody (secondary antibody specificity) and untreated sections (false positive signals due to autofluorescence). The mean fluorescence signal intensity was measured on a single representative cross-section at high magnification using the ImageJ2 Fiji 2.14.0 software (https://imagej.net/ accessed on 20 December 2023).

For each control and treatment replication, 5–6 slices of three plants were used. In the capacity of time points of PEG treatment, there were again chosen 2 h and 3 days after the beginning of PEG treatment. For each time point of PEG exposure, immunohistochemical experiments were performed with three biological replicates. Each replicate included three control and three treatment plants. From each plant, five root segments from the absorption zone were obtained, resulting in a total of 15 segments for both untreated and treated plants. Out of these, five segments each were treated with PIP1 and PIP2 antibodies, while the remaining five segments were assigned to the control and negative control.

### 2.9. Xylem Sap pH Measurements

Measurement of the pH level of the xylem sap was carried out 2 h after the beginning of the water stress effect. Xylem sap was collected from primary roots. To this end, 7-day-old maize plants were taken from the climate chamber and placed into a small cuvette with a small amount of 0.0025 M CaCl_2_ solution. Then, the root was cut off at distance of 2 cm from the grain, and a thin elastic tube 5 cm long with an attached syringe was connected to the cut-off end of the root. Under the effect of the slight vacuum created by the syringe, the xylem sap was collected for 5 min. While collecting the xylem sap, it was difficult to obtain the necessary amount of the sap from one plant to measure the pH using a microelectrode. Therefore, we had to use 5–6 plants for every control and treatment variant. A total of 25 microliters of xylem sap was collected from 5–6 plants for each control and treatment group. pH measurements were carried on using the microelectrode InLab Micro (MetlerToledo, Viroflay, France). Xylem sap pH measurements were conducted in three biological replicates using a total approximately 35 plants, with 15–17 plants for both the control and treatment groups.

### 2.10. Leaf Water Content Measurements

Relative leaf water content was measured at two time points: 2 h and 1 day after plants’ exposure to water stress. In order to measure the relative water content in leaves, the second fully developed leaves were cut and immediately placed into a polyethylene bag and weighed to get the fresh mass M_fr_. After the determination of M_fr_, the samples were places into Petri dishes with distilled water. Then, the samples were incubated for two hours in a vacuum chamber under the pressure of 30 kPa. After blotting the leaves with filter paper, the samples were again put into polyethylene bags, and the mass of the saturated leaves was measured (M_sat_). Then, the samples were dried in a desiccator at 105 °C until a constant weight (M_dr_) was achieved. The relative water content was calculated as (M_fr_ − M_dr_)/(M_sat_ − M_dr_) × 100%. Each control and treatment group consisted of three plants. The experiments were performed in three biological replicates, resulting in a total of 18 plants (9 control and 9 treatment plants) used for each time point (2 h and 1 day of PEG treatment).

### 2.11. Shoot and Root Growth Rate Measurements

The growth rate of roots and shoots was measured dynamically over two hours after the start of the plants’ treatment with PEG. Then, the growth rate was measured for the same plant every day for three days. The growth rate was calculated by dividing the absolute values of the elongation of the whole roots and shoots by the observation time. To determine root and shoot elongation values, the plants were photographed using a camera through the transparent walls of the climate chambers. The obtained images were analyzed using the ImageJ 1.43m program. Six plants were used for each control and treatment group. A total of 36 plants were used for the measurements, which were carried out in three biological replicates.

### 2.12. Statistics

Statistical analysis was conducted using OriginPro 8.5 (OriginLab Corp., Northampton, MA, USA). Normal distribution of the data was assessed using the Kolmogorov–Smirnov and Shapiro–Wilk tests. Statistical analysis of two-factor exposure data was performed using two-factor analysis of variance (two-way ANOVA followed by Tukey test at *p* = 0.05). Statistical analysis of short-term PEG exposure data was performed using one-way analysis of variance (one-way ANOVA followed by Tukey test at *p* = 0.05). Comparison of mean values between the control and treatment groups was performed using the *t*-test at *p* = 0.05. The calculation results are shown in the figures as the mean values and standard deviations. Differences between means were considered significant at *p* < 0.05.

## 3. Results

### 3.1. Dynamics of Intercellular Water Transfer and Cell Membrane Permeability in the Roots of Intact Plants under Water Stress

**Figure 2:**
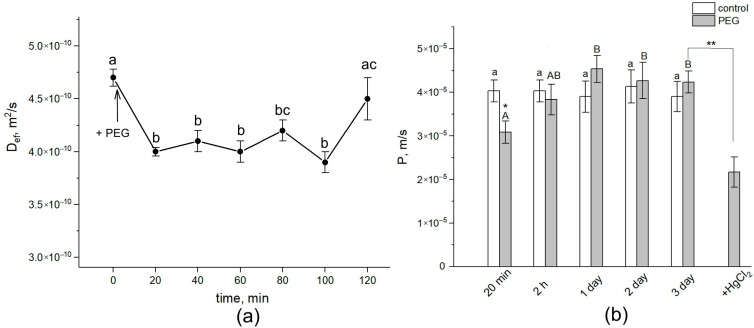
Dynamics of D_ef_ and P in the roots of intact maize plants under water stress. (**a**) Short-term dynamics of D_ef_ at the initial stage of PEG impact. The solid arrow indicates the moment of addition of the 10% PEG solution to the root medium. D_ef_ was measured during the first 120 min of PEG exposure. D_ef_ was measured at a diffusion time of 700 ms. (**b**) Cell water permeability P in the control (white columns) and PEG treatment (gray columns) groups. Bars show ±SD (*n* = 3). For figure (**a**), mean separation was determined by one-way ANOVA (one factor: time of treatment) followed by the Tukey test at *p* = 0.05. The initial value of D_ef_ was considered as the value at zero PEG exposure time. Different letters indicate significant differences between D_ef_ values at different treatment times. For figure (**b**), mean separation was determined by two-way ANOVA (two factors: time and treatment) followed by Tukey test at *p* = 0.05. Different letters indicate significant differences between D_ef_ values at different treatment times. Different lowercase letters correspond to significant differences within the control values, while different uppercase letters correspond to significant differences between treatment values. * Indicates a significant difference between the control and treatment values. ** Indicates a significant difference between *p* values of three-day PEG treatment and the 20-min HgCl_2_ impact after PEG treatment.

During the first 20 min, treatment with the 10% PEG solution to the intact roots of maize in the course of NMR measurements directly in climate chambers resulted in a drastic decrease of approximately 15% in the average water self-diffusion coefficient in roots (D_ef_) in the radial direction of the absorption zone (Figure 2a). 

Diffusional cell water permeability P was reduced by approximately 22% (Figure 2b). D_ef_ and P remained at this level for about 1.5 h. Then, the cell water permeability abruptly increased to values close to the control ones (before PEG treatment). After 24 h, an additional increase in D_ef_ and P was observed. During next two days, the values of D_ef_ and P remained at the level of the initial control. The further treatment of roots with 200 µM of mercuric chloride (aquaporin blocker) resulted in a significant, by 40%, decrease in P. Thus, water stress causes significant changes in the intensity of transcellular water transfer already in the early stages of exposure.

### 3.2. Water Stress Impact on Symplastic Water Transport in the Intact Plant Roots

**Figure 3:**
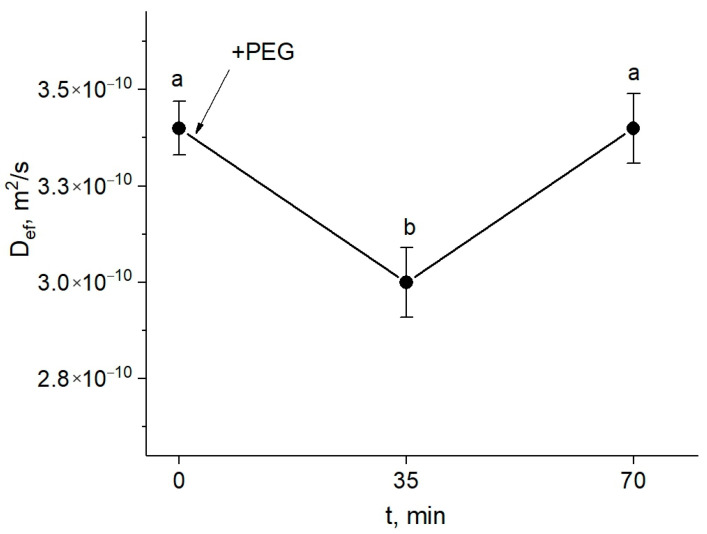
Dynamics of D_ef_ of water in the roots of intact maize plants under water stress in the presence of the paramagnetic complex MnDCTA in the root extracellular space. The solid arrow indicates the time of application of the 10% PEG solution to the roots. D_ef_ was measured at a diffusion time of 700 ms. Bars show ±SD (*n* = 3). Mean separation was determined by one-way ANOVA (one factor: time of treatment) followed by Tukey test at *p* = 0.05. Different letters indicate significant differences between treatment time.

The study of the response of the symplastic water transfer pathway for water deficit plants was carried out using paramagnetic contrasting agents. First, a detailed study was carried out to select the optimal paramagnetic complex at the optimal concentration, in the course of which NMR contrast agents at different concentrations were used, and the rate of symplastic water transfer through plasmodesmata in the presence of these agents in the extracellular space of root tissues was estimated [31]. This approach showed that during the first 30 min of treatment with PEG, the intensity of the symplastic water transfer in roots decreased (Figure 3). 

However, an increase (recovery) in the water transfer parameters was observed, and after approximately one hour, the value of D_ef_ achieved the control level (before the treatment).

### 3.3. Relative Contribution of Radial Water Transport via Root Apoplast under Water Stress

**Figure 4:**
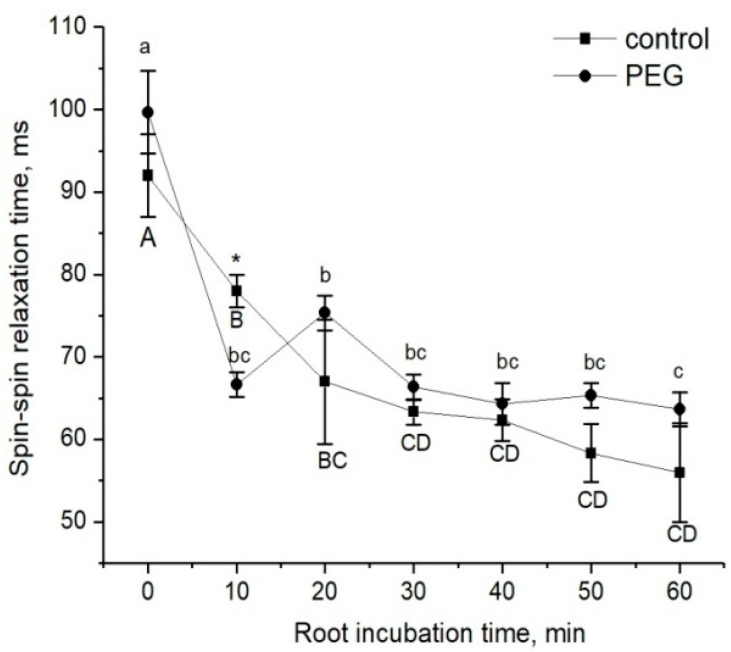
Dynamics of the spin–spin relaxation time T_2_ of water in the apoplast compartment of root tissues during incubation of intact plant roots in the 10 mM MnDCTA solution (solid squares) and in the 10 mMMnDCTA in 10% PEG solution (solid circles). Bars show ±SD (*n* = 3). Mean separation was determined by two-way ANOVA (two factors: treatment and time) followed by Tukey test at *p* = 0.05. Different lowercase letters correspond to significant differences within control values, while different uppercase letters correspond to significant differences between treatment values. * Indicates a significant difference between the control and treatment groups.

The estimation of changes in the apoplastic water transfer in the root radial direction in response to water stress was based on the study of the rate of penetration of the paramagnetic complex MnDCTA (estimated by the NMR method) and fluorescent nanoparticles based on the iron and manganese levels (estimated by the microscopy method), as mentioned in the Materials and Methods. The dynamics of the decrease in the spin–spin relaxation time (T_2_) of the penetration of MnDCTA into the root apoplast is shown in Figure 4. During the time of monitoring, the amplitude of decrease in T_2_ in control samples and samples treated with PEG happened to be approximately the same, and this points to the similar rate of paramagnetic penetration into the apoplast. However, a visible difference in the dynamics of T_2_ changes was observed during the first minutes of treatment with PEG. In particular, after the addition of PEG and MnDCTAto into the root medium, an abrupt and reversible decrease in T_2_ was observed at the beginning, and then the dynamics of the T_2_ decrease became similar to the control samples (Figure 4).

**Figure 5:**
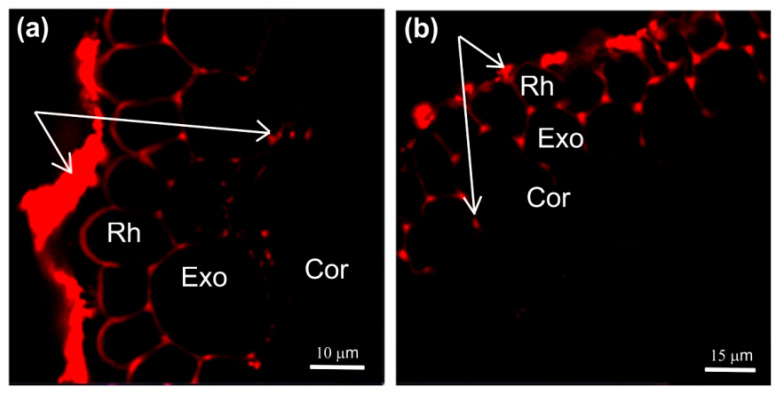

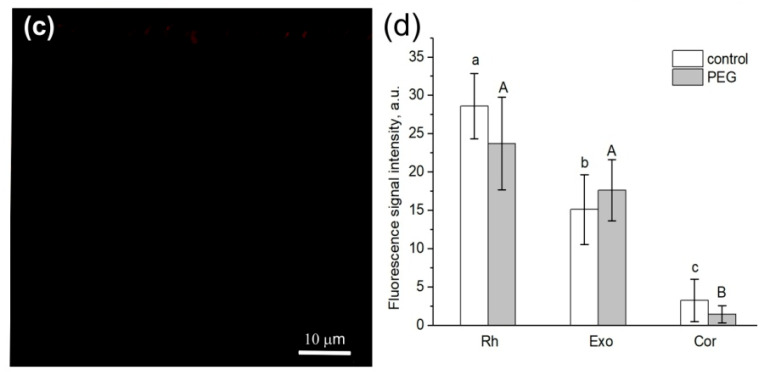
Cross-sections of maize roots from suction zone after 45 min of incubation of roots of intact plants in solutions with fluorescent nanoparticles: (**a**) incubation of roots in an aqueous solution of nanoparticles at a concentration of 6 g/L under normal conditions (without PEG treatment), (**b**) incubation of roots under PEG treatment in a 10% PEG solution with nanoparticles at a concentration of 6 g/L, and (**c**) control (without fluorescent nanoparticles). (**d**) Fluorescence signal intensity of different root tissues. Bars show ±SD (*n* = 5). Mean separation was determined by two-way ANOVA (two factors: treatment and tissue) followed by Tukey test at *p* = 0.05. Different lowercase letters on the top of the columns correspond to significant differences within control values, while different uppercase letters correspond to significant differences between treatment values. Rh, rhizodermis; Exo, exodermis; Cor, cortex. White arrows indicate fluorescent nanoparticles.

The second method of comparative estimation of changes in the apoplast water transfer using fluorescent nanoparticles showed that in the case of 1.5 h of treatment with PEG, the depth of nanoparticle penetration into the root apoplasts of the control and PEG-treated samples was the same (Figure 5).

It should be noted that the application of paramagnetic fluorescent nanoparticles to contrast the pathways of water transport in plant roots was a novel. In general, the abovementioned results indicate that there was no significant decrease in the rate of the apoplastic water transport in maize plant roots during the first 1.5 h of treatment with PEG.

### 3.4. AQP Gene Expression in the Roots and Leaves under Water Stress

**Figure 6:**
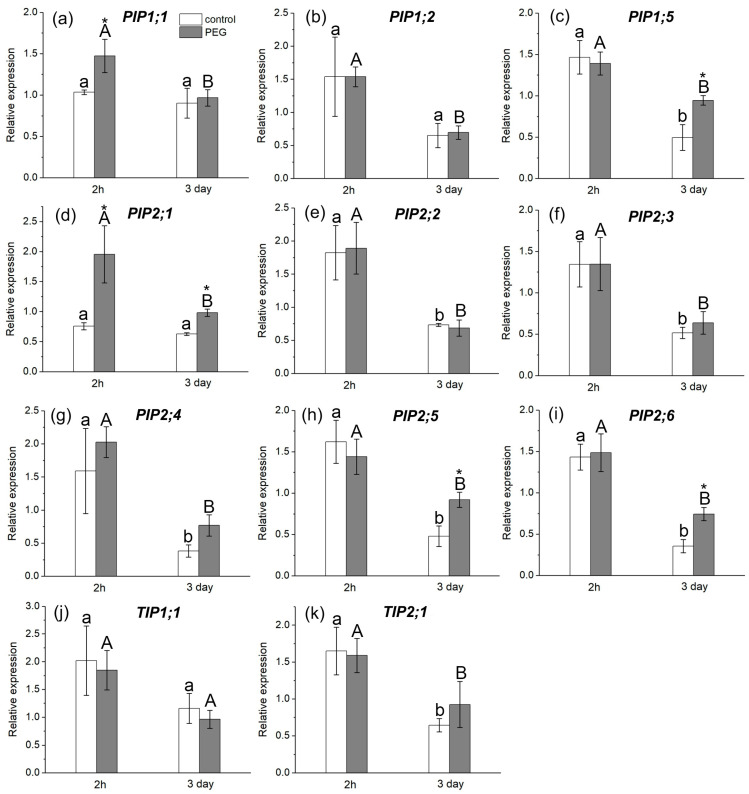
Relative expression levels of PIP and TIP AQP genes in the roots of maize plants in control (without PEG treatment) and after exposure of plant roots to 10% PEG solution for 2 h and 3 days. Bars show ±SD (*n* = 6). Mean separation was determined by two-way ANOVA followed by Tukey test at *p* = 0.05. For each AQP, different lowercase letters on the top of the columns correspond to significant differences within control values, while different uppercase letters correspond to significant differences between treatments. * Indicates significant difference between control and treatment: (**a**) *PIP1;1*; (**b**) *PIP1;2*; (**c**) *PIP1;5*; (**d**) *PIP2;1*; (**e**) *PIP2;2*; (**f**) *PIP2;3*; (**g**) *PIP2;4*; (**h**) *PIP2;5*; (**i**) *PIP2;6*; (**j**) *TIP1;1*; (**k**) *TIP2;1*.

**Figure 7:**
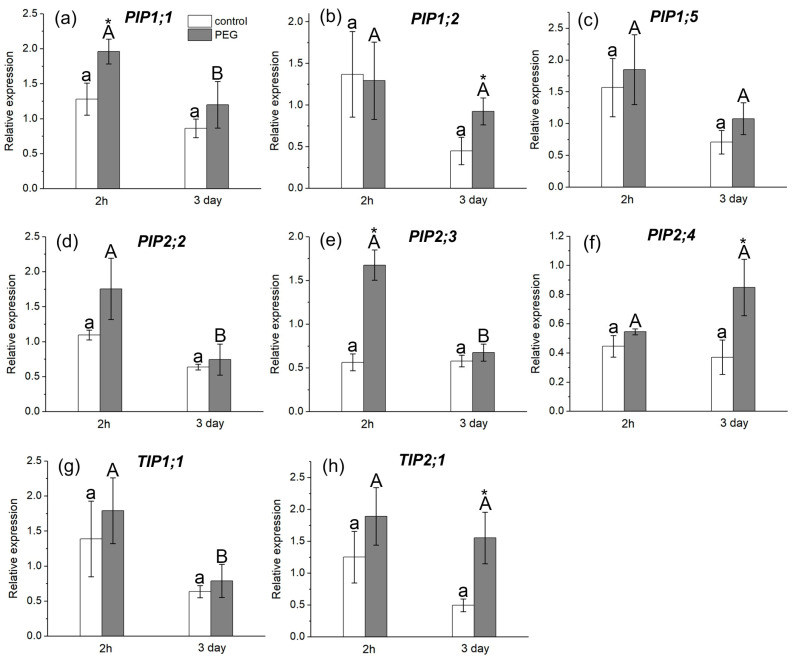
Relative expression levels of PIP and TIP AQP genes in the leaves of maize plants in control (without PEG treatment) and after exposure of plant roots to 10% PEG solution for 2 h and 3 days. Bars show ±SD (*n* = 6). Mean separation was determined by two-way ANOVA (two factors: treatment and time) followed by Tukey test at *p* = 0.05. For each AQP, different lowercase letters on the top of the columns correspond to significant differences within control values, while different uppercase letters correspond to significant differences between treatments. * Indicates significant difference between control and treatment: (**a**) *PIP1;1*; (**b**) *PIP1;2*; (**c**) *PIP1;5*; (**d**) *PIP2;2*; (**e**) *PIP2;3*; (**f**) *PIP2;4*; (**g**) *TIP1;1*; (**h**) *TIP2;1*.

The study of AQP gene expression in response to water stress showed a distinct response in the form of overexpression of some AQP genes in the roots and leaves of maize plants (Figure 6 and Figure 7). In particular, an increase in the relative expression level of *PIP1;1* and *PIP2;1* genes in roots (Figure 6a,d) and *PIP1;1* and *PIP2;3* genes in leaves (Figure 7a,e) was registered 2 h after the beginning of PEG treatment. After three days of PEG treatment, a change in both the quantitative and qualitative composition of AQPs with increased expression levels in the roots and leaves was observed. In particular, an increase in the expression level was observed for *PIP1;5*, *PIP2;1*, *PIP2;5*, and *PIP2;6* AQP genes in roots (Figure 6c,d,h,i) and for *PIP1;2*, *PIP2;4*, and *TIP2;1* AQP genes in leaves (Figure 7b,f,h). 

It is worth noting that, according to the literature, the isoforms *PIP1;5*, *PIP2;1*, *PIP2;4*, *PIP2;5*, and *PIP2;6* are among the genes with the most pronounced level of expression in maize roots, which is consistent with our results [20]. According to our data, the *PIP2;1*, *PIP2;5*, and *PIP2;6* isoforms had very low control levels of expression in maize leaves, which is also consistent with the literature data.

### 3.5. In Situ Immunolocalization of PIP1 and PIP2

**Figure 8:**
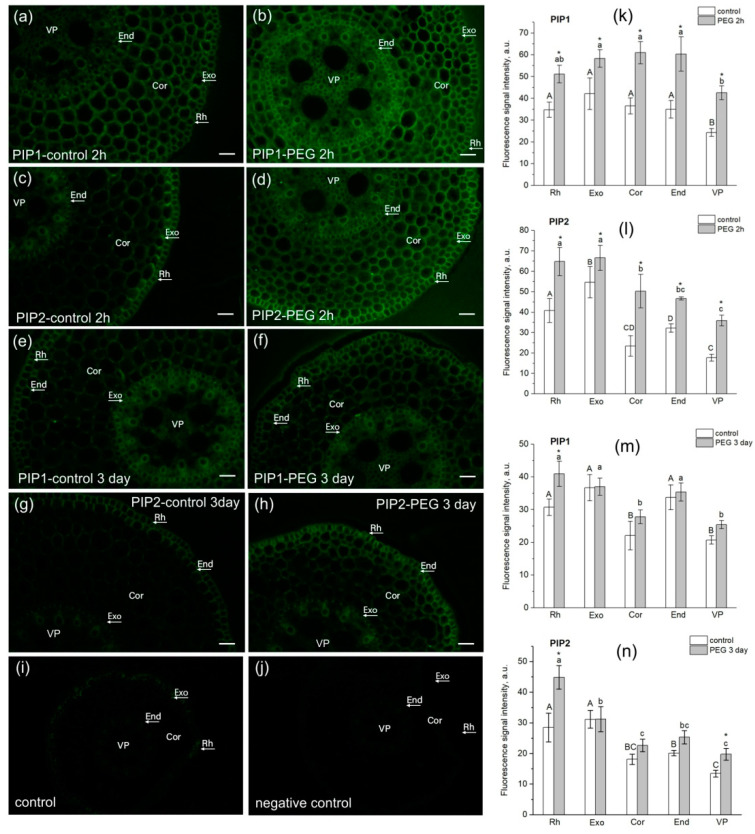
Localization of PIP1 and PIP2 aquaporins in maize root tissues at 3 cm from the root tip in control (without PEG treatment) and after exposure of roots to 10% PEG solution for 2 h and 3 days. (**a**,**b**) Localization of PIP1 in control roots (**a**) and after PEG treatment for 2 h (**b**). (**c**,**d**) Localization of PIP2 in control roots (**c**) and after PEG treatment for 2 h (**d**). (**e**,**f**) Localization of PIP1 in control roots (**e**) and after PEG treatment for 3 days (**f**). (**g**,**h**) Localization of PIP2 in control roots (**g**) and after PEG treatment for 3 days (**h**). (**i**) Control (without antibody treatment of slices). (**j**) Negative control (after treatment of slices with secondary antibodies only). Bars = 35 µm. Rh, rhizodermis; Exo, exodermis; Cor, cortex; End, endodermis; VP, vascular parenchyma. (**k**–**n**) Fluorescence signal intensity of different root tissues in control and after PEG treatment. Mean separation was determined by two-way ANOVA (two factors: tissue and treatment) followed by Tukey test at *p* = 0.05. * Indicates significant difference between control and treatment. Different lowercase letters on the top of the columns correspond to significant differences between untreated tissues, while different uppercase letters correspond to significant differences between treated tissues.

The study of AQP localization in root tissues had several aims. Firstly, it was necessary to confirm the presence of AQPs in the root cortex in the water transport measurement zone for qualitative interpretation of the results of NMR measurements of cell water permeability. Secondly, it was interesting to identify possible differences and changes in the localization of PIP1 and PIP2 AQPs in root tissues under normal conditions and under water stress. In this study, we used antibodies to PIP2 and PIP1 aquaporins and not to individual isoforms. This was due to the fact that often the individual isoforms of the PIP subfamilies do not differ much in localization, especially in roots. For example, Hachez and co-authors showed that the localization of PIP2 isoforms, in particular, PIP2;1/2;2 and PIP2;5, along a cross section of a maize root in the suction zone (5 cm from the root tip) d not differ much [41]. The results of our experiments showed that in the suction zone of maize roots, both PIP1 and PIP2 aquaporins were localized in the exodermis, cortex, and endodermis (Figure 8). 

Upon plants’ exposure to water stress, at both the early (2 h of exposure) and late (3 days of exposure) stages, an increase in the fluorescence signal of PIP1 (Figure 8b) and PIP2 (Figure 8d,h) AQPs was registered in root cortex cells, and in the case of PIP1, also in the endodermis (Figure 8b). In the variant with PIP2, the most marked difference was observed for cortex cells (Figure 8d,h). These data may indirectly indicate an increase in AQPs’ abundance in response to water stress and correlate with the above results on AQP gene expression (Figure 6). It is worth noting that the root stem, including the protoxylem vessels, showed an increase in fluorescence signal intensity in some cases (Figure 8b,d,h). A similar result was obtained in maize plants in the work of Hachez and co-authors (Hachez et al., [41]). This may be due to changes in phloem unloading processes under water stress, which require facilitation of aquaporin-mediated water transport for rapid osmotic potential equilibration.

### 3.6. Transpiration Rate, Plant Growth, Leaf Water Content, and Xylem Sap pH under Water Stress

**Figure 9:**
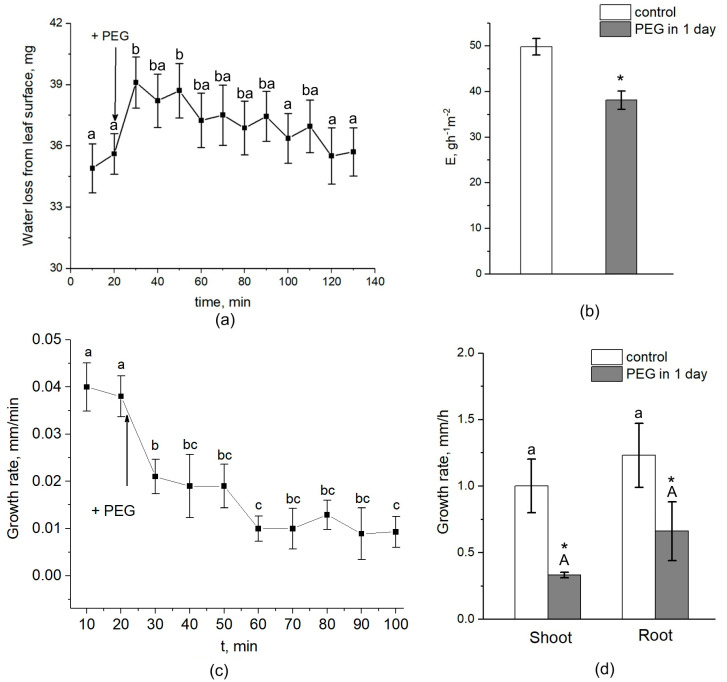
Effect of PEG treatment on transpiration rate and root and shoot growth rate in maize plants. (**a**) Short-term dynamics of water evaporation from leaf surface under exposure of roots to 10% PEG solution. (**b**) Transpiration rate in control (without treatment) (white column) and after 1 day of PEG treatment (gray column). (**c**) Short-term dynamics of root elongation growth under exposure of roots to 10% PEG solution. (**d**) Shoot and root growth rate in control (without treatment) (white column) and after 1 day of PEG treatment (gray column). Bars show ±SD. For (**a**,**c**), mean separation was determined by one-way ANOVA (one factor: time of treatment) followed by Tukey test at *p* = 0.05. The initial values before PEG application to the roots were considered as the values at zero PEG exposure time. Different letterson the graphs (**a**) and (**c**) correspond to significant differences between values at different treatment times. For (**b**), mean separation was determined by two-group *t*-test at *p* = 0.05. For (**d**), mean separation was determined by two-way ANOVA (two factors: treatment and organ) followed by Tukey test at *p* = 0.05. * Indicates significant difference between control and treatment. Different lowercase letters correspond to significant differences within the control values, while different uppercase letters correspond to significant differences between treatment values.

Interestingly, the transpiration rate was increased by approximately 10–15% during the first five minutes after the beginning of PEG treatment and then, after approximately 30–40 min, began to decrease (Figure 9a). After one day of water stress, transpiration rate was 25–30% lower than the control (without PEG treatment) (Figure 9b). Over the next 3 days of measurements, the transpiration rate also remained below the control level. The dynamics of plant root growth under PEG impact are shown in Figure 9c. As can be seen, the treatment of the roots with a 10% PEG solution almost immediately caused a decrease in the rate of root growth. One day after the beginning of plant exposure to water stress, the growth rate of roots and shoots decreased with respect to the control by 35% and 57%, respectively (Figure 9d).

**Figure 10:**
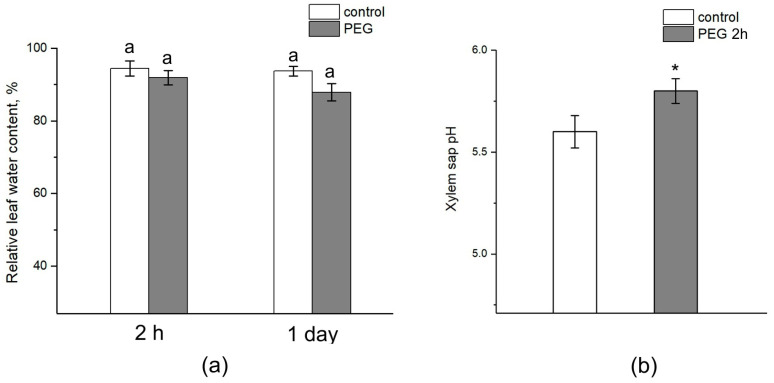
Effect of PEG treatment on relative leaf water content and xylem sap pH. (**a**) Relative leaf water content in control plants (without PEG treatment) (white column) and after 2 h and 1 day of PEG treatment (gray column). (**b**) Xylem sap pH in control plants (without treatment) (white column) and after 2 h of PEG treatment (gray column). Mean separation in figure (**a**) was determined by two-way ANOVA (two factors: time and treatment) followed by Tukey test at *p* = 0.05. No significant differences were found in the relative water content of the leaves. Mean separation in figure (**b**) was determined by *t*-test at *p* = 0.05. The identical letters on the top of the columns indicates no significant differences between values. * Indicates significant difference between control and treatment.

The relative water content in the leaves after 2 h of exposure to PEG did not differ from the control (Figure 10a). After one day, there was a tendency to decrease the relative water content in the leaves of plants subjected to stress, while the relative water content in control plants was unchanged. However, the difference between the control and treated plants was not significant. Interestingly, water stress also caused a shift in the xylem sap pH to the alkaline side. In control samples, the pH level was approximately 5.6, while exposure to PEG for 2 h resulted in an increase in the xylem sap pH to 5.8 (Figure 10b).

## 4. Discussion

In the vast majority of works, in order to characterize the state of the hydraulic system of plants, including ones under the impact of stress factors, the measurement of the hydraulic conductivity of plant organs (roots, stems, leaves) is used [42]. There have been many studies with a traditional cell pressure probe or root pressure chamber methods showing that root hydraulic conductivity changed significantly after osmotic or salt stress [43,44]. Most of these studies showed that plants’ exposure to osmotic stress led to a decrease in root hydraulic conductivity but did not show which pathways of water transport are engaged and at what time stages of exposure this occurs, nor how it correlates with the reactions in the aboveground organs of intact plants. The technique for measuring hydraulic conductivity almost always involves cutting off the part of the plant being studied and is usually carried out after prolonged exposure to stress. In this case, registration of rapid changes in the water transport system and measuring the dynamics of these changes directly under stress conditions and in the native state becomes difficult. The distinctive feature of the present study is that the methodology used made it possible to register the early dynamics of water transport in the roots of intact plants directly under the impact of water stress in controlled environmental conditions. The obtained results differed from the above-mentioned works since they characterize the contribution of different pathways of radial water transport (transcellular, symplast, and apoplast) dynamics.

Plant roots are the first target when plants are exposed to water stress caused by the application of a PEG solution to the root medium when, obviously, the main events during the early stage of stress exposure should occur primarily in the roots. Thus, root exposure to PEG in our experiments initially caused a sharp decrease in D_ef_ and P by approximately 15% and 22%, respectively. At this level, the value of the cell water permeability was maintained for 1.5 h, after which there was an increase (recovery) in water transfer parameters to approximately control values (before PEG application). This suggests rather the involvement of regulatory factors in the process of the adjustment of the root and whole plant hydraulic system to water stress. Aquaporins (AQPs) are known to make a significant contribution to providing and regulating transcellular water transport in roots [9]. Regulation of water transport by AQPs may occur through changes in their activity and/or abundance, and the contribution of these two factors may be different at different stages of the water stress impact [19]. Rapid changes in cell membrane permeability are thought to be associated with the opening/closure of AQPs as a result of changes in their conformation. This mechanism, known as AQP gating, can be realized by chemical, mechanical, and even electrical stimuli [45,46]. Vandeleur et al.’s work on shoot-to-root signaling showed that leaf cutting in soybean and maize plants caused rapid, within a few minutes, changes in turgor pressure and membrane permeability in the root cells [47]. According to the authors, the reason for such rapid changes in membrane permeability can be AQP gating through a mechanical stimulus caused by changes in the cell turgor pressure. In this case, the primary messenger for changes in the cell turgor and subsequent changes in the cell membrane permeability can be a xylem-mediated hydraulic signal caused by a change in the water potential gradient in the plant after leaf cutting. Obviously, the treatment of plant roots by PEG causes a sharp osmotic stress. The emergence of an additional osmotic force disrupts the initially established equilibrium of water transport in the root. This is indirectly supported by our results of the NMR experiments on the study of the dynamics of spin–spin relaxation times (T_2_) of water magnetization in the roots during incubation of the roots of intact maize plants in a 10 mM MnDCTA solution (Figure 4). Whereas in control plants the T_2_ of the apoplast water during the penetration of the paramagnetic agents into the root apoplast decreased uniformly from the beginning, in the case of PEG application to the roots simultaneously with MnDCTA, there was first a sharp and reversible decrease in T_2_ in the sample registered within the first 10 min. It is difficult to relate this effect to any physiological function. Most likely, it is a consequence of the disruption in the water balance between the intracellular and extracellular water compartments of the root surface layer under acute exposure to PEG. Then, the dynamics of T_2_ became the same as in the control. Interestingly, there was a sharp and reversible change in the intracellular component of relaxation attenuation. Given the rapidity and reversibility of the reaction at the initial stage of PEG impact, it can be argued that this reaction is related not only to the penetration of MnDCTA into the root apoplast. An additional reason is most likely to be a fast change in the water exchange between the intracellular and extracellular compartments of the root tissues, which is also reflected in the magnetic relaxation times of water.

Drought and salinity stress reduce the osmotic potential in the root environment, leading to cellular water loss, reduced turgor, and cell damage. One plant strategy that can be initiated against osmotic stress is osmoregulation by accumulating organic osmolytes (such as soluble sugars and proline) and mineral ions such as sodium in their cells [48]. Although this process can be initiated at the onset of stress, it can take a significant amount of time to reach the required concentration of osmolytes and ions. Thus, a reliable increase in the concentration of osmolytes has been recorded in various plants, including maize, several days after the onset of osmotic stress action [49].

It can be assumed that PEG treatment stimulates the primary outflow of water predominantly from the root apoplast, since the resistance of the apoplast to water flow is significantly less than the resistance of cell membranes. The initial change in the apoplast’s water potential and the water content of the cell walls can be a mechanical stimulus for closing of aquaporins through the AQP gating mechanism. In this case, aquaporins closing may represent a protective function that prevents water loss by root cells under osmotic stress conditions. Another reason for the decrease in the intensity of transcellular water transport during the first minutes of PEG treatment, which also cannot be excluded, may be a physical relationship between apoplastic and transcellular water transport or, in other words, the influence of the rate of apoplastic water transport on the rate of transcellular water transport and vice versa. Crossing the plasmalemma and entering the cell wall along the transcellular pathway, water molecules are influenced by the apoplastic water flow. It is logical to assume that the rate of apoplastic water transport will influence the rate at which water molecules can enter the next cell, completing another transcellular transition. Then, the rate of apoplastic water transport and its changes, for example, under osmotic stress impact, should be reflected in the rate of transcellular water transport. It is difficult to say what the nature and degree of physical influence of one water transport pathway on another should be. This issue deserves special attention but, most likely, is associated with experimental difficulties in solving it.

If the rapid decrease in water permeability of root cells at the initial stage of water stress is caused primarily by physical or mechanical factors, then the recovery of water permeability, observed after 2 h of PEG treatment, is largely associated with molecular and genetic regulatory factors. This is supported, in particular, by upregulation of *PIP1;1* and *PIP 2;1* AQP genes in the roots after 2 h of root treatment with PEG (Figure 6a,d). These AQP isoforms are among those that provide the greatest contribution to water transport in maize roots [41]. It is possible that the increase in the transcript level of these AQPs has a compensatory effect, due to which the diffusional permeability, which was reduced at the early stage of water stress, is restored. Besides transcription, cycling of AQP proteins also affects their abundance in a certain membrane system. For instance, PIP internalization has been observed under salt stress [43,50]. In addition, AQP activity (open state) can be increased by its phosphorylation [51]. The amount of up-regulated AQPs was higher at the later stage of water stress impact. Exposure of roots to mercuric chloride (inhibitor of AQPs) on the third day of PEG treatment significantly reduced the cell water permeability P (by 40%) (Figure 2b). This reduction in P under the influence of mercuric chloride is usually observed in control maize plants [30]. This means that under water stress conditions, the abundance and/or activity of AQPs increases.

It is interesting that in this case, the water permeability of root cells did not differ much from the control, but the total water absorption by the roots and the transpiration rate decreased. This indicates a decrease in the intensity of apoplastic water transport and its contribution to the total water transport in the roots at the later stages of water stress impact. Considering the possible interaction between transcellular and apoplastic water transport pathways, it can be assumed that even with an increase in AQPs’ abundance and/or activity, the cell water permeability will not be able to increase proportionally due to a decrease in the rate of apoplastic water flow and the state of water in the cell wall. The effect of long-term exposure to water stress on AQPs’ gene expression and root hydraulic conductivity has some contradictory results. Thus, water deficiency or drought can cause either a decrease in AQPs’ transcript levels and cell water permeability, especially under severe water stress [52,53,54], or an increase [55,56]. It is assumed that the contribution of both types of reactions can vary depending on water stress intensity and duration, but it is not completely clear what is the triggering factor for switching from one type of reaction to another. Often, changes in the root hydraulic conductivity do not correlate with changes in the water permeability of membranes in root cortex cells. For example, while a significant increase in cell hydraulic conductivity of PEG-treated cortical cells in maize roots was measured, no such effect was observed at the whole root level [55]. Similar independent responses were reported for the grapevine *Chardonnay* cultivar during a long-term drought period, in which the cortex cell hydraulic conductivity increased while the root hydraulic conductivity decreased [7]. The above disagreements are often associated with species and genetic differences in plants, with different plant growing conditions, as well as with the involvement of additional stress factors and plant damage during the measurement of plant hydraulic conductance. In our study, exposure of intact maize plants to moderate water stress only led to the overexpression of AQP genes, and there were no variants with a decrease in AQP expression at either the early or late stages of exposure. In addition, immunolocalization of PIP1 and PIP2 AQPs indirectly indicated an increase in AQPs’ abundance in root tissues (Figure 8). Thus, the obtained results indicate that the strategy of adjustment of a plant’s hydraulic system to water stress is aimed at increasing the contribution of AQPs and, in general, transcellular pathways in the regulation of water transport in roots.

There are no experimental data in the literature on the effect of water stress on the intensity of symplastic water transport via plasmodesmata. The model of explicit cross-section hydraulic anatomy (MECHA), which calculates the flow of water through the cell walls, membranes, and plasmodesmata of each individual cell along a cross section of the root, predicts that root hydraulic conductivity is very sensitive to the aperture of plasmodesmata, despite their presumably low conductivity [39]. Using the spin-echo NMR and paramagnetic doping, we tried to estimate the response of symplastic water transport in the roots of intact plants to water stress. As it turned out, the symplastic pathway in the roots also responded to PEG treatment (Figure 3). Moreover, the nature of its response was approximately the same as that of the transcellular pathway. During the first minutes of stress impact, the rate of symplastic water transport decreased, and then it restored to the control level. It was methodically difficult to carry out longer measurements of the dynamics of symplastic transfer over several hours and days. Thus, the symplastic pathway can also contribute to the total hydraulic conductivity of the roots under water stress. It is not yet clear whether the plasmodesmata aperture changes under the influence of water stress. Further experiments, for example, using the inhibitor of callose synthesis 2-deoxy-d-glucose, can help bring some clarity to this issue. Interestingly, the response of the water transport in roots, which was expressed in a sharp and reversible decrease in the transcellular and symplastic water transport (Figure 2 and Figure 3), was accompanied by an equally sharp but shorter-term increase, by approximately 10–15 percent, in the rate of transpiration (Figure 9a). First, this confirms the existence of a functional connection between water conductance in roots and stomatal conductance in leaves. Second, the rate of registered changes suggests that this functional coupling must be mediated by fast signals. Given the high response rate, one such signal could be a hydraulic signal. As a result of the sharp slowdown in the water supply to the root upon PEG application, when the transpiration rate is still maintained at a normal level, a short-term wave of rarefaction can occur in the root xylem. This wave may represent a hydraulic (mechanical) signal that can propagate very fast from roots to leaves and trigger a chain of further reactions there, providing a coordinated response of underground and aboveground plant organs to water stress. The change in the water potential gradient and hydraulic conductivity of roots can also be a direct regulator of transpiration rate. In favor of this, for example, are the results of experiments with rhizosphere sealing, in which external overpressure was applied to the root medium [57]. It was shown that variation in the external pressure applied to the roots was necessarily reflected in the stomatal conductance. Transpiration is known to be the driving force behind passive water transport through the root apoplast. An increase in the transpiration rate during the first few minutes of PEG exposure can maintain the rate of apoplast transport at the initial stage of water stress exposure. The results of experiments using fluorescent nanoparticles suggest that the rate of apoplastic water transport in the root remains unchanged during the first few minutes of PEG exposure. Both control and PEG-exposed roots showed the same depth of nanoparticle penetration in the root apoplast during the same observation time (Figure 5). Additionally, the total decrease in spin–spin relaxation times of water during the penetration of the paramagnetic complex MnDCTA into the root apoplast was approximately the same in both the control and experimental samples (Figure 4). It can be assumed that the purpose of the short-term increase in transpiration is the accelerated delivery of hormones, in particular ABA, as well as other signaling molecules from the roots to increase their concentration in the leaves. After application of PEG to the roots, the increased transpiration rate was maintained for an average of 25–30 min (Figure 9). In some experiments, this time could reach 40 min. During this time, the concentration of hormones could reach the necessary level to trigger molecular and genetic mechanisms of regulation of stomatal conductance mediated, in particular, by AQPs. It is assumed that ABA accumulation in the stomata guard cells can activate AQPs in the plasma membrane [58]. In our case, AQPs in the leaves and roots were clearly responsive to water stress. After 2 h of water stress, an increase in the expression level of *PIP1;1*, and *PIP2;3* was observed in the leaves (Figure 7a,e). After three days of PEG treatment, the expression of these isoforms did not differ from the control, but there was an increase in the expression of other AQPs, *PIP1;2, PIP2;4*, and *TIP2;1* (Figure 7f,h). Interestingly, *PIP1;1* showed high activity simultaneously in both the roots and leaves, but only at an early stage of the stress impact. This may emphasize its specific role in the short-term regulation of water transport under stress conditions. The upregulation of *TIP2;1* is also worth noting. The role of PIPs in the stress response is well established in most cases, but some reports specifically point out the role of TIPs under water deficient conditions. For example, in rice, the expression of *OsTIP1;1* was up-regulated in roots and shoots in response to water stress [59]. There are also many conflicting results in the literature regarding the expression of AQP genes in leaves under water stress. For example, Alexandersson and coauthors investigated the expression of AQPs in *Arabidopsis* in response to drought stress and showed that all PIP genes were downregulated in response to drought stress in leaves except *AtPIP1;4* and *AtPIP2;5*, which were upregulated. Moreover, *AtPIP2;6* and *AtSIP1;1* were constitutively expressed and were not significantly affected by the drought stress [52]. The expression of *AtPIP2;5* was also significantly upregulated in leaves in response to a combination of drought and heat stress [60]. In our experiments, the over-expression of AQPs in leaves after 2 h of plant exposure to water stress coincided with a decrease in the transpiration rate, indicating the involvement of AQPs in the regulation of stomatal conductance. Moreover, according to the literature data, AQPs can play a dual role in this regulation simultaneously. The first role consists of the accelerated transport of hydrogen peroxide into stomata guard cells to activate the membrane system, followed by the outflow of ions from the cells. The second function is most likely associated with an increase in water outflow from the stomata guard cells, resulting in a decrease in their turgor pressure and stomata closure. It is worth noting that the above effects were also accompanied by an increase in the xylem sap pH (Figure 10b). Recently, there has been increasing evidence that changes in pH can be an important factor in signaling systems and in the coordination of hydraulic system components at the whole plant level during plants’ adaptation to stress. For example, Liang Fang and co-authors showed that changes in the stomatal conductance, root and leaf hydraulic conductance, and AQP gene expression in tomato plant roots under increasing atmospheric CO2 concentration were accompanied by changes in the xylem sap pH [61]. The change in pH can both act as an activator of hydrogen peroxide synthesis and directly affect the activity of AQPs via phosphorylation [45]. Thus, the change in xylem sap pH under water stress can be an additional regulatory factor. It should be noted that the overexpression of AQPs in leaves and decreased transpiration rate were still present three days after the onset of water stress. At the same time, there was also a reduction in the root and shoot growth rate (Figure 9c,d). In general, this indicates the maintenance of plants’ adjustment strategies aimed at preventing water loss through transpiration by inducing stomatal closure under prolonged exposure to water stress. Thus, overexpression of AQP genes in leaves can be aimed at increasing plant resistance to water stress. The results of some studies are in favor of this. For example, under conditions of a severe soil water deficit, maize *PIP2;5* OE lines can preserve the ability of roots to absorb water and the hydraulic conductivity of leaves [58]. Transgenic tobacco plants over-expressing the wheat TaAQP7 were found to be more drought tolerant compared to non-transgenic tobacco plants [62]. Transgenic *Arabidopsis* plants expressing *PIP1* from *Vicia faba* also showed increased drought tolerance [63].

## 5. Conclusions

The results of the present work, obtained using an original methodological and technical approach that allowed for the simultaneous measurement of water transport characteristics in the roots and transpiration rates in the leaves of intact plants under strictly controlled environmental parameters, indicated a distinct response of the components of the plants’ hydraulic system to water stress, both in the early and in the later stages of stress exposure. Often, due to methodological and technical limitations, early signals and responses during the first minutes of stress impact are ignored. However, as the results of this work show, these signals and responses, in the form of a decrease in the transcellular water transfer and short-term increase in transpiration, form late responses and probably determine the strategy of plants’ adjustment to water stress. It was shown that under conditions of water deficiency, transcellular water transport in the roots becomes dominant. Symplastic water transport also responds to water stress. An important role of root and leaf AQPs in the process of adjustment of plants’ hydraulic systems to stress effects has been shown. It is suggested that an additional factor in the root-to-shoot coordination can be a change in the xylem sap pH. Taking into account the developed network of AQP distribution in plant organs and tissues and their important role in the regulation of water flows, it can be assumed that AQPs contribute by providing simultaneous regulation of water transport processes in roots and shoots under water stress. It should be noted that the non-destructive spin-echo NMR method used in the present study, together with additional technical equipment in the form of climatic chambers for intact plants and in combination with other physiological and molecular genetic methods, represents a promising tool and can be used in studies of water transport in plants and mechanisms of coordination of plant hydraulic system components under various types of abiotic stresses.

## Data Availability

Data will be provided upon request.

## Supplementary Materials

The following supporting information can be downloaded at: https://www.mdpi.com/article/10.3390/cells13020154/s1, Table S1. Primers for target PIP and TIP aquaporin genes and three reference genes (*ZmFPGS*, *ZmMEP*, *ZmUBCP*).
