# Supplementary material for: Real-Time Dynamics of Water Transport in the Roots of Intact Maize Plants in Response to Water Stress: The Role of Aquaporins and the Contribution of Different Water Transport Pathways
Source: Cells. 2024 Jan 15;13(2):154. doi: 10.3390/cells13020154 (PMC10814095; doi:10.3390/cells13020154)
Supplement: Supplementary file 1 [file cells-13-00154-s001.zip › cells-2767444-supplementary.pdf]

Supplementary

Table S1. Primers for target PIP and TIP aquaporin genes and three reference genes (ZmFPGS, ZmMEP, ZmUBCP).

Gene	Primer name	Sequence 5' -> 3'	Accession number
PIP1;1	PIP1;1_F	gccgtaatttacaaccagcac	00001d002690
	PIP1;1_R	acctactcttgaacgggatcg	
TIP2;1	TIP2;1_F	accgtcgtcgtttgcttttg	00001d051362
	TIP2;1_R	tgaatggaccagggcaaagg	
PIP2;5	PIP2;5_F	ctccttcagccgctagatcg	00001d003006
	PIP2;5_R	ctgtccttgtccagcctctg	
PIP2;1	PIP2;1_F	agttcgctgccaaggactac	00001d019563
	PIP2;1_R	gccaccgtgatgtacaggaa	
PIP1;5	PIP1;5_F	gccagttcagctagccatca	00001d051872
	PIP1;5_R	caccgtaccaaaacccaagc	
PIP2;2	PIP2;2_F	aaagaaaggccttcgctgga	00001d005421
	PIP2;2_R	tctccccacgctagatcgat	
PIP2;6	PIP2;6_F	tcaggtcaagtgttgcagct	00001d019565
	PIP2;6_R	cacacttgctttccacaccg	
TIP1;1	TIP1;1_F	gctcagcttagctccactcc	00001d027652
	TIP1;1_R	ttgatcggcattttcacggc	
PIP1;2	PIP1;2_F	tgttggcaccttcatcctagt	00001d017526
	PIP1;2_R	cagtggggcaaggatagg	
PIP2;3	PIP2;3_F	tgttggcccctcttccgattg	00001d051174
	PIP2;3_R	ccattggtcgtcccaggc	
PIP2;4	PIP2;4_F	gttccggtgctagctcctctc	00001d017288
	PIP2;4_R	caggccttgtccttgttgtaga	
ZmFPGS	FPGS_F	atctcgttggggatgtcttg	00001d048514
	FPGS_R	agcaccgttcaaatgtctcc	
ZmMEP	MEP_F	tgtactcggcaatgctcttg	00001d018359
	MEP_R	tttgatgctccaggcttacc	
ZmUBCP	UBCP_F	caggtggggtattcttggtg	00001d001913
	UBCP_R	atgttcgggtggaaaacctt	
